# HrrF Is the Fur-Regulated Small RNA in Nontypeable *Haemophilus influenzae*

**DOI:** 10.1371/journal.pone.0105644

**Published:** 2014-08-26

**Authors:** Estevan A. Santana, Alistair Harrison, Xinjun Zhang, Beth D. Baker, Benjamin J. Kelly, Peter White, Yunlong Liu, Robert S. Munson

**Affiliations:** 1 The Center for Microbial Pathogenesis, The Research Institute at Nationwide Children's Hospital, Columbus, Ohio, United States of America; 2 The Biomedical Genomics Core, The Research Institute at Nationwide Children's Hospital, Columbus, Ohio, United States of America; 3 The Center for Microbial Interface Biology, The Ohio State University, Columbus, Ohio, United States of America; 4 Department of Pediatrics, The Ohio State University, Columbus, Ohio, United States of America; 5 School of Informatics and Computing, Indiana University, Bloomington, Indiana, United States of America; East Carolina University School of Medicine, United States of America

## Abstract

Nontypeable *Haemophilus influenza*e (NTHi) are Gram-negative commensal bacteria that reside in the nasopharynx. NTHi can also cause multiple upper and lower respiratory tract diseases that include sinusitis, conjunctivitis, bronchitis, and otitis media. In numerous bacterial species the ferric uptake regulator (Fur) acts as a global regulator of iron homeostasis by negatively regulating the expression of iron uptake systems. However in NTHi strain 86-028NP and numerous other bacterial species there are multiple instances where Fur positively affects gene expression. It is known that many instances of positive regulation by Fur occur indirectly through a small RNA intermediate. However, no examples of small RNAs have been described in NTHi. Therefore we used RNA-Seq analysis to analyze the transcriptome of NTHi strain 86-028NP*rpsL* and an isogenic 86-028NP*rpsL*Δ*fur* strain to identify Fur-regulated intergenic transcripts. From this analysis we identified HrrF, the first small RNA described in any *Haemophilus* species. Orthologues of this small RNA exist only among other *Pasteurellaceae*. Our analysis showed that HrrF is maximally expressed when iron levels are low. Additionally, Fur was shown to bind upstream of the *hrrF* promoter. RNA-Seq analysis was used to identify targets of HrrF which include genes whose products are involved in molybdate uptake, deoxyribonucleotide synthesis, and amino acid biosynthesis. The stability of HrrF is not dependent on the RNA chaperone Hfq. This study is the first step in an effort to investigate the role small RNAs play in altering gene expression in response to iron limitation in NTHi.

## Introduction

Bacteria experience numerous types of stress during their growth. Environmental stressors range from nutrient availability, changes in pH, temperature variations, redox fluctuations, and osmolarity changes. However, bacteria have developed responses that alter their gene expression in order to cope with the many stresses encountered [1]. Understanding the gene expression changes bacteria undergo in response to environmental stresses, particularly the changes in gene expression undergone by pathogens, can lead to the identification of therapeutic targets that may prevent or treat disease.

The majority of stress responses occur at the transcriptional and post-transcriptional level. The main form of transcriptional regulation occurs through the use of specific sigma factors that alter RNA polymerase promoter specificity [2]. Gram-negative and Gram-positive bacteria utilize sigmaS and sigmaB, respectively, as part of the general stress response to cope with entry into stationary phase [3], [4], [5]. In response to envelope stress Gram-negative bacteria utilize sigmaE to regulate the expression of proteins involved in the degradation and/or refolding of misfolded proteins in the periplasm [6], [7].

In addition to the use of sigma factors, bacteria have evolved regulatory networks that use DNA-binding transcription factors that respond to specific stresses. In numerous bacteria, iron stress is controlled by the ferric uptake regulator (Fur) [8], [9]. In the presence of its corepressor Fe(II), Fur binds to a conserved sequence of DNA designated as the Fur box and blocks transcription of target genes. However, Fur also positively affects gene expression. Fur can directly bind DNA and activate gene expression in certain bacterial species [10]. However, most examples of positive gene regulation by Fur are indirect and mediated through a small regulatory RNA (sRNA) [11], [12].

The most studied Fur-regulated sRNA is RyhB from *Escherichia coli* [13]. Under high iron conditions Fur and its corepressor Fe(II) bind to a Fur box located in the *ryhB* promoter and so blocks RyhB's transcription. Conversely, during low iron conditions, Fur does not bind the *ryhB* Fur box and so allows transcription of RyhB. In certain cases RyhB positively regulates gene expression by disrupting target mRNA secondary structure, thus allowing translation to occur, for example *shiA* in *E. coli* [11], [14], [15]. However, most examples of RyhB regulation described to date depend on RyhB base pairing with its RNA target followed by the degradation of both the target mRNA and RyhB itself [16]. Thus, through negative regulation by RyhB, Fur acts indirectly to positively affect gene expression. RyhB orthologues have been identified in several species of related enteric pathogens including *Shigella, Salmonella, Yersinia,* and *Klebsiella* [17], [18], [19], [20], [21], [22]. RyhB orthologues have also been identified in non-enteric species, for example *Pseudomonas aeruginosa, Vibrio cholera, Neisseria meningitidis, Neisseria gonorrhoeae,* and *Azotobacter vinelandii* [23], [24], [25], [26], [27], [28]. Although numerous bacterial species contain Fur orthologues, Fur-regulated sRNAs have not been identified in all bacteria that contain Fur.

Nontypeable *Haemophilus influenzae* (NTHi) is a Gram negative commensal residing in the nasopharynx. However, when NTHi gains access to niches outside of the nasopharynx, it exists as a pathogen [29], [30]. For example, in the middle ear NTHi it is a major cause of otitis media (OM). Previous studies have shown that NTHi upregulates expression of iron uptake systems in the middle ear, indicating that the middle ear environment is relatively iron and/or heme-poor. [31], [32], [33]. This iron and/or heme restricted environment requires NTHi to quickly adapt in order to survive [29].

Because Fur-regulated sRNAs have been shown to play an important role in iron homeostasis in other bacteria we investigated whether NTHi encoded Fur-regulated sRNAs. Furthermore, we wanted to know how Fur-regulated sRNAs in NTHi influence gene regulation. To accomplish our goals we first generated a *fur* mutation in NTHi strain 86-028NP*rpsL* and used RNA-Seq to analyze the entire transcriptome of both the 86-028NP*rpsL* and 86-028NP*rpsL*Δ*fur* strains. We searched for intergenic transcripts whose expression increased in the absence of Fur. From this analysis we identified a single Fur-regulated and iron-responsive ∼97nt sRNA that we designated *hrrF* which is conserved among the *Pasteurellaceae*. Moreover, in strain NTHi 86-028NP and closely related NTHi strains, there is a minor *hrrF* transcript which contains an extended 3′ region resulting in a transcript of ∼260nt designated *hrrF_L_.* An *hrrF* deletion was generated in a strain containing a *fur* mutation to generate NTHi 86-028NP*rpsL*Δ*fur*Δ*hrrF_L_.* RNA-Seq was used to identify transcripts whose abundance was responsive to the presence or absence of HrrF. We identified six transcripts whose abundance was altered when *hrrF* was deleted in the *fur* mutant background which indicated that *hrrF* targets these mRNAs. An additional study demonstrated that the stability of HrrF was independent of the presence or absence of Hfq.

OM is the most frequently diagnosed illness in children in the United States [34]. As such it is the number one cause for antibiotic prescription among this patient population [34]. This is of great concern due to the possibility of the emergence of antibiotic resistant strains of bacteria. Therefore is it imperative that we find ways to prevent NTHi disease. Understanding how NTHi fine-tune their gene expression during low iron conditions via sRNAs will enhance our understanding of the survival strategies during this stress condition with the potential to identify new therapeutic targets for disease treatment and prevention.

## Methods

### Bacterial strains, culture media used, and growth conditions

Strains and plasmids used in this study are listed in Table S1. NTHi strain 86-028NP was isolated from the nasopharynx of a child with chronic OM [35] and is well characterized *in vitro* and *in vivo* in the chinchilla model of OM [29], [35], [36], [37]. The genome sequence is published [38]. For routine culturing, strains were grown on Chocolate II agar plates (Fisher Scientific, Waltham, MA) or on chocolate agar plates containing antibiotics as needed. The final antibiotic concentrations employed were 200 µg spectinomycin/ml, 20 µg kanamycin/ml, or 1000 µg streptomycin/ml. For routine liquid culture, strains were grown in brain heart infusion medium supplemented with 2 µg heme/ml and 2 µg β-Nicotinamide adenine dinucleotide/ml (sBHI). Except where indicated, RNA-Seq experiments and experiments in which iron concentrations were manipulated were performed in an iron-depleted, defined iron source medium (DIS), a modification of defined growth media designed by Hasen *et al.* and Coleman *et al.* [31], [39], [40]. Cells were iron-depleted for approximately 13 hours of static growth in DIS at 37°C, 5% CO_2_. Iron-depleted cells were then transferred to DIS supplemented with 10 µg human hemoglobin/ml and grown to mid-logarithmic phase at 37°C, with shaking at 50 rpm. For iron chelation studies, cells were then split into two aliquots and 2,2′-bipyridine was added to one aliquot to a final concentration of 500 µM. After 15 minutes of growth at 37°C with shaking at 50rpm, cells were removed for RNA isolation. Where indicated, iron sulfate was then added to cultures at a final concentration of 200 µM. Cells were removed for RNA isolation at 0, 5, 10, and 20 minutes after iron sulfate addition. To determine that iron chelation had no effect on cell viability, cells were also removed from each aliquot, serially diluted, and enumerated on chocolate II agar plates. For RNA stability studies, after growth to mid-logarithmic phase in DIS, cells were then split in two and rifampicin was added to a final concentration of 250 µM. Growth was continued at 37°C with shaking at 50rpm. At 0, 2, 4, 6, 10, 20, and 30 minutes after rifampicin addition cells were removed for RNA isolation.

### Construction of nonpolar deletion mutations in strain 86-028NP*rpsL* protein-encoding genes

Mutant strains were generated in the strain 86-028NP*rpsL* background using the method of Carruthers et al [37], [41], [42]. Mutants were selected on medium containing spectinomycin. The spectinomycin antibiotic resistance cassette was subsequently removed by the activity of the Flp recombinase. After removal of the spectinomycin resistance cassette additional mutations were generated in the desired genetic backgrounds using the same methodology. Primers used in mutant generation are listed in Table S2.

### Construction of strains with deletions in the *hrrF* coding region

Mutations in the *hrrF* region were generated as above with the following exceptions. Three hundred and twenty base pairs of the intergenic region containing *hrrF_L_* starting seven base pairs downstream of the NTHI1320 (*pmbA*) stop codon were deleted to generate the 86-028NP*rpsL*Δ*hrrF_L_* strain. The HrrF region in strain 86-028NP has an extended 3′ region not found in the HrrF region of the genomes of many members of the *Pasteurellaceae*. In order to determine the significance of this 3′ extension, we generated a strain lacking the extended 3′ region. Primers ES252 and ES254 were used to amplify the pGEM-T Easy-*hrrF_L_* construct excluding the 3′ portion of *hrrF_L_*. The resulting PCR product was phosphorylated using the End-It DNA Repair kit (Epicentre, Madison, WI) and self-ligated followed by transformation into *E. coli* DH5α. The resulting plasmid, pGEM-T Easy-Δ*hrrF*3′, maintained the 1kb of flanking DNA sequence and so was linearized and naturally transformed via the MIV method [43] into 86-028NP*rpsL*Δ*hrrF_L_*::spec-*rpsL.* Recombination of the Δ*hrrF*3′ construct into this strain resulted in the loss of the spec-*rpsL* cassette. Transformants were selected for growth on streptomycin. Primers used in *hrrF_L_* and *hrrF*3′ mutant constructions are listed in Table S2.

### Construction of complemented mutant strains

Construction of pT-*fur* was described previously [44]. To construct pT-*hfq* the coding sequence of *hfq* was PCR amplified with primers that generated an NdeI site 5′ of *hfq* and a BamHI site 3′ of *hfq.* The resulting PCR product was then cloned between the NdeI and BamHI sites in pT. pT-*hfq* was then electroporated into strain 86-028NP*rpsL*Δ*hfq.* Primers used to construct complementation plasmids are listed in Table S2. Expression of Hfq from pT-*hfq* was verified via western blot using anti-*E. coli* Hfq antibody (a kind gift from Gisela Storz).

### RNA isolation and rRNA removal

Strain 86-028NP*rpsL* was grown in sBHI at 37°C, with shaking at 180rpm. Alternatively, strains were grown in DIS supplemented with 10 µg human hemoglobin/ml. At mid-logarithmic phase, cells were collected for RNA-isolation. Total RNA was isolated via the hot phenol method as previously described [45] with one exception. The culture-phenol mixture was heated at 90°C for 10 minutes in order to merge the two phases. rRNA was removed using the Ribo-Zero kit (Gram negative) (Epicentre, Madison, WI) following the manufacturer's protocol. RNA integrity and rRNA removal were confirmed with an RNA Nano Chip on the Agilent 2100 Bioanalyzer (Agilent Technologies, Santa Clara, CA).

### Preparation of RNA-Seq libraries and sequencing

cDNA library construction was carried out using the TruSeq RNA Sample Prep Kit (Illumina, San Diego, CA) according to manufacturer's protocols. The final DNA libraries were validated with the Agilent 2100 Bioanalyzer using the Agilent High Sensitivity DNA Kit. The library concentrations were determined by Q-PCR using KAPA SYBR Fast qPCR kit (Kapa Biosystems, Wilmington, MA). The libraries generated from NTHi 86-028NP*rpsL* grown in sBHI as well as the first biological replicate of strains 86-028NP*rpsL,* 86-028NP*rpsL*Δ*fur*, and 86-028NP*rpsLΔfur*(pT-*fur*) were then run on a Single End flowcell on the HiSeq 2000 (Illumina) and 51bp reads were generated. Biological replicates two and three of strains 86-028NP*rpsL,* 86-028NP*rpsL*Δ*fur*, and 86-028NP*rpsLΔfur*(pT-*fur*) were run on Paired End flowcells, using 2×101bp reads. The second RNA-Seq experiment containing cDNA libraries generated from strains 86-028NP*rpsL,* 86-028NP*rpsLΔfur,* 86-028NP*rpsLΔfurΔhrrF_L_*, and 86-028NP*rpsLΔfurΔhrrF*3′ were run on the HiSeq 2000 (Illumina) on Paired End flowcells, using 2×101bp reads.

### Sequence mapping and quantification of transcript levels

The sequenced reads from the RNA-Seq experiment of the library of sBHI-grown cells were mapped to the NTHi strain 86-028NP genome (NC_007146) using Burrows-Wheeler Aligner (BWA version 0.5.10). The resulting bam file is available on the Sequence Read Archive (SRA) [SRP042147]. Subsequent to our initial RNA-Seq experiment all regions of intergenic expression were annotated (Table S3). From this analysis a BED file was generated from which all future RNA-Seq experiments were aligned using BWA, version 0.78, allowing up to 2 mismatches for read lengths of 50bp or 4 mismatches for read lengths of 100bp. The resulting bam files from the RNA-Seq analysis of 86-028NP*rpsL,* 86-028NP*rpsLΔfur,* and 86-028NP*rpsLΔfur*(pT-*fur*) strains are available on SRA (SRP042197). Reads with multiple best hits were reported with only one mapping location. On average, 95% of the total reads were mappable. The total number of reads corresponding to each annotated region was determined with NGSUtils software (version 0.5.5) [46]. Differential gene expression analysis across different strains was conducted using the Bioconductor package edgeR [47]. Batch effects among biological replicates were accounted for during analysis. The differential gene expression analysis data discussed in this publication have been deposited in NCBI's Gene Expression Omnibus (GEO) [48] and are accessible through GEO Series accession number GSE8891.

### Primer Extension

Primer ES188 was labeled with a VIC fluorescent tag and used to generate 1^st^ strand cDNA from total RNA. Five micrograms of total RNA and 100pmol of ES188 were mixed in a total volume of 30 µl and denatured at 90°C for three minutes then slow cooled to 30°C. Six microliters 0.1M DTT, four microliters Superscript II (Invitrogen, Grand Island, NY), 12 µl 1^st^ strand buffer, 1.5 µl 25mM dNTP (Invitrogen, Grand Island, NY), two microliters RNasin (Ambion, Grand Island, NY), and 0.5 µl RNase free H_2_O were then added. The mixture was incubated at 42°C for 2 hours to generate cDNA. RNA was degraded by adding 10 µl 1M NaOH and heating at 70°C for 10 minutes. Ten microliters of 1M HCl was then added to neutralize the reaction. The cDNA was purified using the MinElute PCR Purification Kit (Qiagen, Valencia, CA). Primer extension analysis was performed at the Plant-Microbe Genomics Facility at The Ohio State University (www.pmgf.osu.edu).

### 5′ rapid amplification of cDNA ends (RACE) analysis

A unique 50bp sequence not present in the NTHi 86-028NP genome was amplified from pLS88 using primers ES133 and ES160 for use in *in vitro* transcription [49]. The resulting PCR product was purified using phenol-chloroform extraction. The 50bp DNA template was used in an *in vitro* transcription reaction with T7 RNA polymerase (New England Biolabs, Ipswich, MA) to generate the RNA adapter according to the manufacturer's protocol. The resulting *in vitro* transcribed RNA was purified by acid phenol-chloroform extraction. Treatment of RNA with Tobacco Acid Phosphatase (Epicentre, Madison, WI), RNA ligation of RNA adapter, 1^st^ strand cDNA synthesis with ES168, 2^nd^ strand cDNA synthesis with ES168 and ES172, and cloning of cDNA were all conducted according to the protocol of Argaman *et al*. [50].

### Northern Blot Analyses

Northern blots were performed using the DIG Northern Blot Starter Kit (Roche, Indianapolis, IN). The RNA probes were generated using T7 RNA polymerase from the DIG RNA Labeling Kit (Roche, Indianapolis, IN) and the DNA template generated from primers ES173 and ES174 (*hrrF*) or ES89 and ES90 (5S rRNA). Northern blots were developed using CDP-*Star* ready-to-use solution (Roche, Indianapolis, IN).

### RNA half-life calculation

Strains were grown as stated for the RNA stability studies. Total RNA ranging from 75ng to 1000ng was probed on the same blots used for probing RNA collected after the addition of rifampicin to determine the dynamic range of the densitometer. Densitometry and half-life calculation was performed on a GS-800 Calibrated Densitometer (Bio-Rad, Hercules, CA).

### Fur pull-down assay

The Fur pull-down assay was performed as previously described [44]. Biotin labeled oligonucleotides containing the putative Fur box sequences were generated using biotin labeled primers as listed in Table S2. These putative Fur box sequences were then scrambled using an automated sequence mixer (http://molbiol.ru/eng/scripts/01_16.html) originally part of the Sequence Manipulation Suite curated by P. Stothard [51]. After scrambling the putative Fur box sequences, the resulting oligonucleotide sequences were checked via Virtual Footprint [52] to ensure a putative Fur box had not been generated. Overlap extension PCR [53] using PCR1 and PCR2 (Table S2) was then used to generate a biotin-labeled oligonucleotide containing the scrambled Fur box sequence.

### Construction of strains containing scrambled Fur boxes 5′ of *hrrF* in the 86-028NP*rpsL* genome

Three predicted Fur boxes upstream of *hrrF* were individually scrambled using an automated sequence mixer (http://molbiol.ru/eng/scripts/01_16.html). All three Fur boxes were also simultaneously scrambled. Overlap extension PCR [53] using primers listed in Table S2 were used to generate DNA fragments containing the scrambled Fur box with 1kb of flanking DNA. The resulting PCR products were transformed into 86-028NP*rpsL*Δ*hrrF_L_*::spec-*rpsL* using the MIV method [43], thus reintroducing *hrrF* back into the genome with the corresponding scrambled Fur box. Removal of the spec-*rpsL* cassette by MIV transformation of PCR products containing scrambled Fur boxes restored streptomycin resistance, allowing for selection of transformants.

### qRT-PCR

qRT-PCR was performed as described in Harrison *et al.* [44].

## Results

### RNA-Seq identification of intergenic transcription in NTHi strain 86-028NP*rpsL*

Trans-encoded small regulatory RNAs in bacteria are generally transcribed from intergenic regions throughout the genome [54]. In order to survey these regions of intergenic transcription in NTHi strain 86-028NP*rpsL* we performed deep sequencing of a cDNA library generated from RNA depleted of ribosomal RNA. This RNA-Seq experiment was conducted as an initial screen to identify all potential sRNAs expressed in 86-028NP during mid-logarithmic growth in sBHI medium. In total there were 166,106,713 aligned reads accounting for 95.6% of the total reads. Using the Integrated Genomics Viewer [55], [56], we identified 86 regions of intergenic transcription and 16 regions of potential antisense transcription. In addition to intergenic transcription there were ∼50 instances of extended 5′ and 3′ UTRs.

From our initial analysis, a BED file was generated which included all annotations contained in strain 86-028NP's original genome annotation [38] plus coordinates for every region of intergenic and antisense transcription identified via RNA-Seq. This new BED file was used for all subsequent RNA-Seq alignments. A spreadsheet containing all gene annotations and all coordinates of intergenic transcription are available in Table S3.

### Identification of a Fur-regulated sRNA

To identify Fur-regulated sRNAs we constructed an unmarked non-polar deletion of *fur* in the NTHi 86-028NP*rpsL* background. We then complemented the *fur* mutation with pT-*fur* [44], which contains the *fur* open reading frame downstream of the *tet* promoter in plasmid pT to generate strain 86-028NP*rpsL*Δ*fur*(pT-*fur*). This and future RNA-Seq experiments utilized strains grown in DIS medium to ensure the concentration and source of iron/heme was defined. Total RNA was collected from the NTHi strains 86-028NP*rpsL*, 86-028NP*rpsL*Δ*fur*, and 86-028NP*rpsL*Δ*fur*(pT-*fur*). cDNA libraries were then sequenced on the Illumina HiSeq 2000 platform.

**Figure 1:**
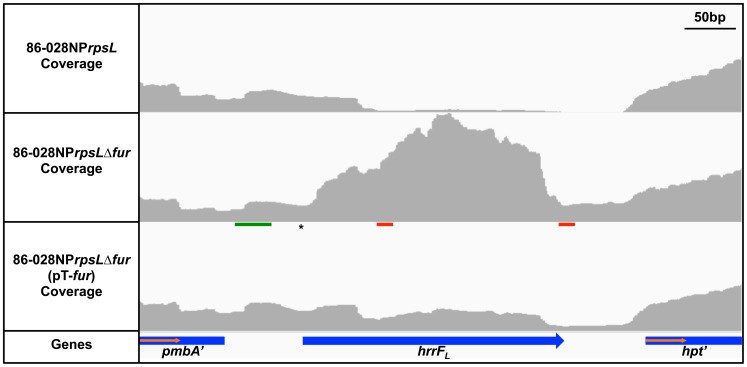
HrrF is upregulated in an 86-028NP*rpsL*Δ*fur* background. Total RNA was isolated from strains 86-028NP*rpsL*, 86-028NP*rpsL*Δ*fur,* and 86-028NP*rpsL*Δ*fur*(pT-*fur*) grown in (DIS) to mid-logarithmic phase at 37°C with shaking at 50rpm. After rRNA removal, cDNA libraries were generated for RNA-Seq analysis on the Illumina HiSeq2000. An intergenic transcript was identified between NTHI1320 (*pmbA*) and NTHI1321 (*hpt*). A representative image using the Integrated Genomics Viewer is shown. The transcript was upregulated in the 86-028NP*rpsL*Δ*fur* background compared to the parent strain. Upregulation of the intergenic transcript was reversed by complementation of the *fur* mutant strain. The vertical axes represent a read depth ranging from 0-1800 reads. The Fur binding site (green line), transcriptional start site (*), and predicted Rho-independent terminators (red lines) are indicated.

An increase in transcription from the intergenic region between NTHI1320 (*pmbA,* encoding a predicted protease) and NTHI1321 (*hpt,* encoding hypoxanthine-guanine phosphoribosyltransferase) was observed in the 86-028NPΔ*fur* mutant compared to the parent (Fig. 1). This increase in the amount of transcript in the *fur* mutant strain was reversed when the *fur* mutation was complemented. The increased amount of transcript in the 86-028NP*rpsLΔfur* strain compared to the parent strain was confirmed by qRT-PCR (Fig. S1).

There are two putative Rho-independent terminators in the intergenic region between *pmbA* and *hpt*. The sequence of the 23nt stem-loops are identical except for a “C” to “T” change at nucleotide 22 of the first stem-loop structure (Fig. 1). The only transcriptional start site (TSS) located in the *pmbA-*hpt intergenic space was identified 5′ to the first terminator via primer extension; a fluorescently labeled cDNA product was compared to a DNA sequencing reaction to determine the last base of the cDNA oligonucleotide (Fig. S2). This base is located 63bp downstream of the *pmbA* stop codon (Fig. 1). 5′ RACE was also used to identify the TSS. By 5′RACE, the TSS is located 65bp downstream of the *pmbA* stop codon, which is in good agreement with the TSS determined by primer extension.

The transcript located in the intergenic space between *pmbA* and *hpt* beginning at the TSS and terminating at the first Rho-independent terminator has been named *hrrF,* for *Haemophilus* regulatory RNA responsive to iron (Fe). A second product corresponding to the region from the same TSS to the second terminator was designated *hrrF*_L_. No other putative sRNA identified in this study demonstrated Fur-dependent regulation.

### HrrF is conserved among *Pasteurellaceae*

**Figure 2:**
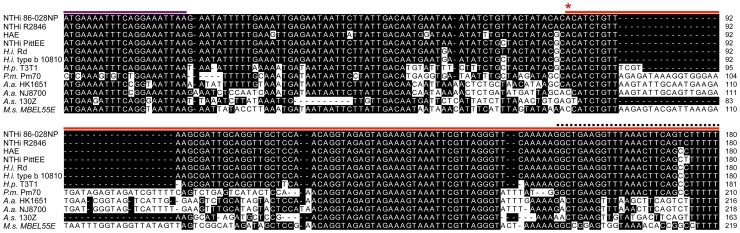
HrrF is conserved among the *Pasteurellaceae.* *hrrF* sequences from 12 *Pasteurellaceae* species were aligned using CLUSTALW. The purple bar indicates the coding sequence for *pmbA.* The red bar indicates the sequence for *hrrF.* The asterisk indicates the TSS. The black dotted line indicates the location of the Rho-independent terminator. Nucleotides that match 86-028NP sequence are shaded black. NTHi: nontypeable *Haemophilus influenzae,* HAE: *H. influenzae* biogroup *aegyptius*, *H.i.*: *Haemophilus influenzae, H.p.*: *Haemophilus parainfluenzae, A.a.* HK1651: *Aggregatibacter actinomycetemcomitans, A.a.* NJ8700: *Aggregatibacter aphrophilus, A.s.: Actinobacillus succinogenes, M.s.: Mannheimia succiniciproducens*

The ∼260nt sequence that encompassed the *hrrF*_L_ region was used in BLAST analysis. There was no sequence similarity to RyhB, PrrF, or NrrF, the iron-responsive sRNAs from *E. coli*, *P. aeruginosa*, and *Neisseria* respectively. There was however, strong sequence conservation of HrrF within the *Pasteurellaceae* (Fig. 2, Fig. S4). For all instances where an *hrrF* orthologue could be identified, a putative Fur box was found via Virtual Footprint [52] analysis upstream of and in close proximity to *hrrF.* Most organisms contained an HrrF orthologue of ∼97bp while some of the distantly related *Pasteurellaceae* had an orthologue of ∼126nt. Next, from the representative *Pasteurellaceae* orthologues of *hrrF* we selected, the entire intergenic region between *pmbA* and *hpt* was aligned using CLUSTALW. The genomic organization of *hrrF* is also well conserved among the *Pasteurellaceae.* The one exception among the selected *Pasteurellaceae* is in *H. influenzae* biogroup *aegyptius* F3031 (HAE). This region is more complex with the *hafABCDE* gene cluster (formerly *hif1* locus, required for the formation of hemagluttinating pili) immediately downstream of *hrrF* and 5′ to *hpt*. The alignment of *hrrF* among the selected *Pasteurellaceae* is shown in Figure 2. An outline of the sequence alignment for the entire intergenic region containing *hrrF* of the selected *Pasteurellaceae* species is presented in Figure S3. The full alignment of the intergenic region between *pmbA* and *hpt* is shown in Fig. S4. As many strains do not have the 3′ portion of the ∼260nt region of *hrrF* present in strain 86-028NP and due to functional data presented later, we have defined the *hrrF* gene as the sequences between the TSS and the end of the first putative transcriptional terminator. Reference to the ∼260nt HrrF transcript present in strain 86-028NP will be referred to as *hrrF_L_*.

### HrrF expression is dependent on iron concentration

**Figure 3:**
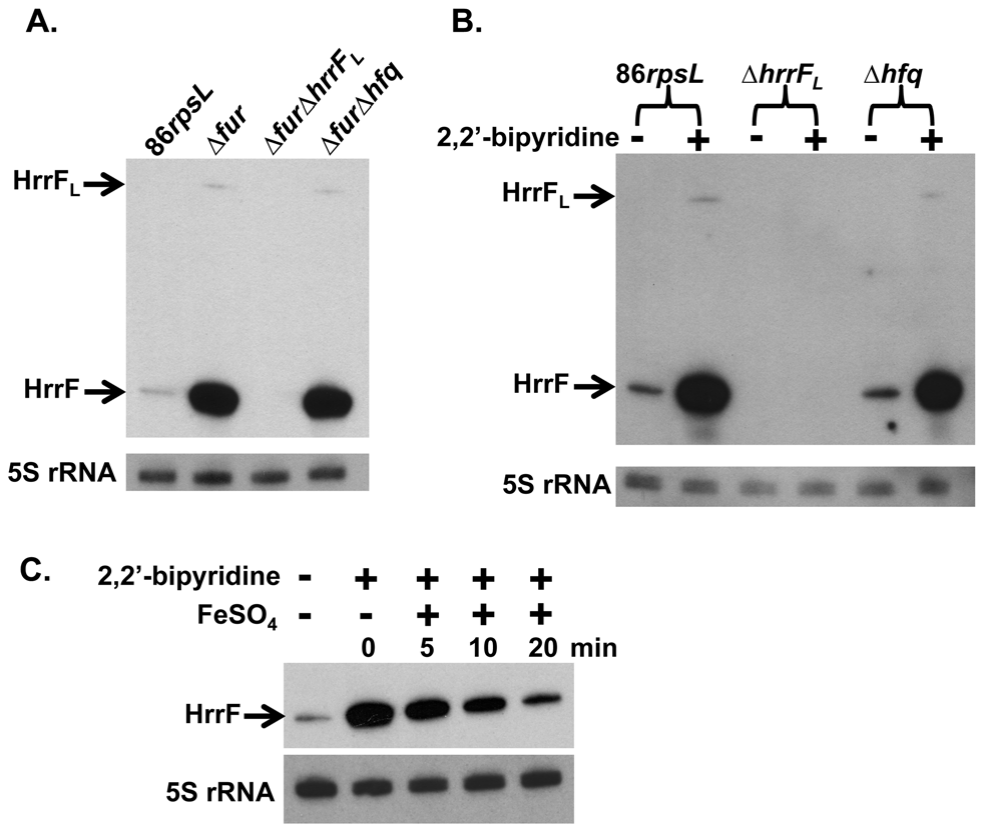
HrrF expression is iron-responsive and Fur-dependent. **A.** Total RNA was purified from strains 86-028NP*rpsL*, 86-028NP*rpsL*Δ*fur*, 86-028NP*rpsL*Δ*fur*Δ*hrrF_L_,* and 86-028NP*rpsL*Δ*fur*Δ*hfq* grown in DIS to mid-logarithmic phase at 37°C with shaking at 50rpm. Northern blots were performed using an RNA probe specific for HrrF. The parent strain produced low levels of the ∼97nt HrrF transcript. Strain 86-028NP*rpsLΔfur* produced HrrF transcript at a greater level than the parent strain. The ∼260nt HrrF_L_ transcript was only produced at detectable levels in the 86-028NP*rpsL*Δ*fur* strain. Transcript levels of HrrF produced in the 86-028NP*rpsL*Δ*furΔhfq* strain were similar to that produced in the 86-028NP*rpsL*Δ*fur* strain indicating that Hfq was not required for HrrF stability. **B.** Total RNA was collected from strains 86-028NP*rpsL,* 86-028NP*rpsL*Δ*hrrF_L_,* and 86-028NP*rpsL*Δ*hfq* grown as in (**A**). At mid-logarithmic phase cultures were split in two and 2,2′-bipyridine was added to one aliquot at a final concentration of 500 µM for 15min. Total RNA was then collected and used for northern blot analysis. HrrF transcript levels increased upon iron chelation. The ∼260nt HrrF_L_ transcript was only detectable upon addition of 2,2′-bipyridine. HrrF transcript levels produced in the 86-028NP*rpsL*Δ*hfq* strain were similar to parental HrrF levels after iron chelation indicating that Hfq was not required for HrrF stability. HrrF transcript was not detectable in RNA from *the hrrF_L_* mutant strain demonstrating the RNA probe's specificity. **C.** NTHi 86-028NP*rpsL* was chelated with 2,2′-bipyridine as in (**B**). After 15 minutes of chelation FeSO_4_ was added to the chelated culture for 20 minutes. Culture aliquots were collected for total RNA extraction before chelation, after 15 minutes of chelation, and 5, 10, and 20 minutes after the addition of FeSO4. HrrF transcript levels decreased over time in response to iron addition. All blots were stripped and probed again with an RNA probe specific for the 5S rRNA to serve as a loading control.

In order to confirm our RNA-Seq data we performed northern blot analysis using an RNA probe specific to HrrF. Strains used in northern blot were first grown in DIS and total RNA was collected for further analysis. When strain 86-028NP*rpsL* was grown in iron replete medium, low levels of HrrF transcripts were detected. However, when the 86-028NP*rpsL*Δ*fur* mutant strain was grown under the same conditions, the amount of HrrF greatly increased (Fig. 3A). The ∼260nt HrrF_L_ transcript could only be detected when Fur repression was relieved. qRT-PCR analysis of *hrrF* expression confirmed that HrrF transcript increased ∼80-fold in the 86-028NP*rpsL*Δ*fur* strain when compared to the parent strain (Fig. S1). To confirm the specificity of the HrrF RNA probe, *hrrF_L_* was deleted in the 86-028NP*rpsL*Δ*fur* mutant background to generate an 86-028NP*rpsL*Δ*fur*Δ*hrrF_L_* double mutant. No *hrrF* transcript was detected in this strain confirming the RNA probe's specificity (Fig. 3A).

The increased abundance of HrrF transcript in the 86-028NP*rpsL*Δ*fur* strain compared to the parent showed that HrrF expression was Fur-regulated and therefore likely dependent on intracellular iron levels. Previous analysis of strain 86-028NP gene expression demonstrated that known Fur-regulated genes are activated in response to iron chelation [44]. Therefore, in order to confirm HrrF dependence on iron levels we grew strains 86-028NP*rpsL* and 86-028NP*rpsL*Δ*hrrF_L_* in DIS followed by iron chelation. As predicted, HrrF transcript levels increased after iron chelation. As with the northern analysis of the *fur* mutant, HrrF_L_ could only be detected when Fur repression was relieved by addition of an iron chelator (Fig. 3B).

To confirm that increases in HrrF transcript after chelation with 2,2′-bipyridine were indeed due to chelation of iron specifically, we grew 86-028NP*rpsL* in DIS chelated with 2,2′-bipyridine. HrrF levels were greatly increased by chelation with 2,2′-bipyridine. After 15 minutes of chelation, FeSO_4_ was added to the chelated cultures to investigate the effect that excess iron addition had on *hrrF* expression. After addition of FeSO_4_, HrrF transcript levels decreased over time as determined by northern blot (Fig. 3C). This result confirmed that *hrrF* expression is indeed responsive to iron levels.

### Fur binds upstream of HrrF

**Figure 4:**
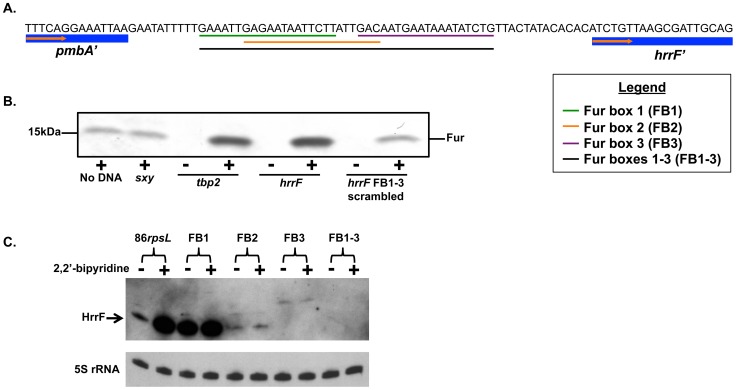
Fur binds upstream of the *hrrF* promoter. **A.** Location of the three predicted Fur boxes in the *hrrF* promoter region. Fur boxes 1, 2, 3, and 1-3 are indicated by the green, orange, purple, and black lines respectively. **B.** Soluble protein fractions from 86-028NP*rpsL*Δ*fur*(pT-*fur*) (+) or 86-028NP*rpsL*Δ*fur* (-) were prepared. Biotinylated oligonucleotides containing the promoter regions of *sxy, tbp2,* and *hrrF* were incubated for 30 minutes with the protein fractions followed by pull-down with streptavidin-agarose beads. Beads were washed, and the bound proteins resolved on a 4.0 to 20.0% SDS-polyacrylamide gradient gel, transferred to nitrocellulose, and probed with anti-*Pseudomonas* Fur antibody. Fur was enriched in the pulldown with the *hrrF* promoter oligo compared to negative controls which contained no oligo, the *sxy* promoter, or the scrambled Fur boxes 1–3 (FB 1–3). Fur binding to the *hrrF* promoter oligo was similar to Fur binding to the promoter of the Fur-regulated gene *tbp2*. The absence of Fur detection with oligonucleotides incubated with 86-028NP*rpsL*Δ*fur* extracts demonstrated the specificity of the anti-Fur antibody. **C.** Strains were constructed in which the predicted *hrrF* Fur box sequences, shown in (**A**), were scrambled in the NTHi 86-028NP*rpsL* genome. Strains were grown in DIS medium to mid-logarithmic phase and split in two. 2,2′-bipyridine was added to one aliquot to a final concentration of 500 µM for 15 minutes. Total RNA was collected from both cultures. Northern blots were performed using an RNA probe specific to HrrF. Blots were stripped and probed with an RNA probe specific to the 5S rRNA as a loading control. Scrambling the Fur box 1 sequence disrupted Fur regulation, but transcription was maintained at parental levels. Scrambling the Fur box 2 or 3 sequence, nearly abolished *hrrF* transcription, suggesting that these putative Fur boxes overlap the *hrrF* promoter. Scrambling Fur boxes 1–3 simultaneously completely abolished *hrrF* transcription. FB = Fur box.

The data presented thus far, have indicated that *hrrF* expression is Fur-regulated and is responsive to iron levels. Virtual Footprint [52] identified three putative Fur boxes that lay within a 41bp region upstream of the *hrrF* TSS (Fig. 4A). All three Fur boxes had strong homology to strain 86-028NP's consensus Fur box [44]. Previously, we demonstrated via a Fur pull-down strategy that in strain 86-028NP, Fur binds to the promoter regions of known Fur-regulated genes [44]. Therefore, we again used a Fur pull-down to show that Fur binds to the *hrrF* promoter.

PCR products were generated using biotin labeled primers that amplified the *hrrF* promoter region. The biotinylated *hrrF* promoter region was incubated with cytoplasmic fractions from either 86-028NP*rpsL*Δ*fur* or 86-028NP*rpsL*Δ*fur*(pT-*fur*) followed by incubation with streptavidin-agarose beads. After rigorous washing the bead-associated proteins were then transferred to a nitrocellulose membrane for immunoblot analysis of the pull-down fraction using an anti-*Pseudomonas* Fur antibody (a kind gift from Michael Vasil). A mock pull-down in which no biotinylated DNA was added to the pull-down reaction served as a negative control for non-specific Fur binding to the streptavidin-agarose beads. Additionally, the biotinylated promoter region for *sxy*, a promoter known not to contain a Fur box was used as a negative control. Performing the Fur pull-down using the 86-028NP*rpsL*Δ*fur* strain and its corresponding complemented strain ensured that the protein band detected via immunoblot was indeed Fur.

When the *hrrF* promoter was present in the assay, Fur binding was enriched as compared to the negative control promoter or the no DNA control (Fig. 4B). The amount of Fur bound to the *hrrF* promoter was similar to that of Fur bound to the known Fur-regulated gene *tbp2.* Carpenter *et. al* demonstrated the binding specificity of Fur in *Helicobacter pylori* by scrambling the Fur box of Fur-regulated genes [57]. Therefore, to show that Fur binding to the Fur boxes in the *hrrF* promoter was specific, the pull-down assay was repeated using a biotin labeled promoter region in which the Fur box sequences were scrambled [44], [57]. When the three Fur boxes were scrambled (FB1-3), enrichment of Fur in the pulled-down fraction was lost (Fig. 4B).

To further demonstrate that Fur binds the *hrrF* promoter at the predicted Fur boxes, we scrambled the predicted Fur boxes upstream of *hrrF* in the strain 86-028NP*rpsL* genome. HrrF expression was then assayed via northern blot of RNA from strains grown under iron replete or iron depleted conditions. When the entire 41bp predicted Fur-binding region that begins 11bp downstream of the *pmbA* stop codon was scrambled (Fur Boxes 1–3), no *hrrF* transcript could be detected (Fig. 4C). Similarly, when the predicted Fur box closest to the *hrrF* TSS (Fur box 3) was scrambled, *hrrF* transcription was nearly abolished. However, altering the Fur box 3 sequence increased the size of the detected HrrF transcript by ∼20nt. It's possible that scrambling the Fur box 3 sequence artificially altered the promoter region of *hrrF* resulting in an altered TSS. Fur box 2 is predicted to be 3′ of Fur box 1 but overlaps Fur box 1 by 13bp. When Fur box 2 was scrambled, *hrrF* transcription was reduced but not abolished completely (Fig. 4C). Finally, when the most 5′ Fur box (Fur box 1) was scrambled, transcription of HrrF occurred at parental levels during both iron replete (compare lanes 2 and 4), and iron restricted conditions (compare lanes 1 and 3). This result further supports our hypothesis that HrrF is Fur-regulated. In addition, Fig. 4C clearly showed that Fur was not able to bind to the scrambled Fur binding site present at Fur box 1.

### HrrF stability and half-life are not dependent on Hfq

In numerous bacteria Hfq acts as an RNA chaperone involved in a majority of sRNA-target interactions [58], [59]. Without Hfq, the stability of many sRNAs are altered [16]. To determine whether steady-state levels of HrrF were dependent on the RNA chaperone Hfq we deleted *hfq* in the 86-028NP*rpsL*Δ*fur* background, generating an 86-028NP*rpsL*Δ*fur*Δ*hfq* strain. Analysis of *hrrF* transcript levels via northern blot demonstrated that there was no detectable difference in the amount of *hrrF* transcript produced by the 86-028NP*rpsL*Δ*fur* and 86-028NP*rpsL*Δ*fur*Δ*hfq* strains indicating that steady-state levels of HrrF were not dependent on Hfq (Fig. 3A). We further showed that Hfq does not affect steady state transcript levels of HrrF by assessing how iron chelation in the absence of Hfq affected *hrrF* transcription. After iron chelation, the HrrF transcript level was similarly increased in both the 86-028NP*rpsL*Δ*hfq* strain and the parent as determined by northern blot analysis (Fig. 3B). Again, HrrF steady-state transcript levels were not dependent on Hfq.

**Figure 5:**
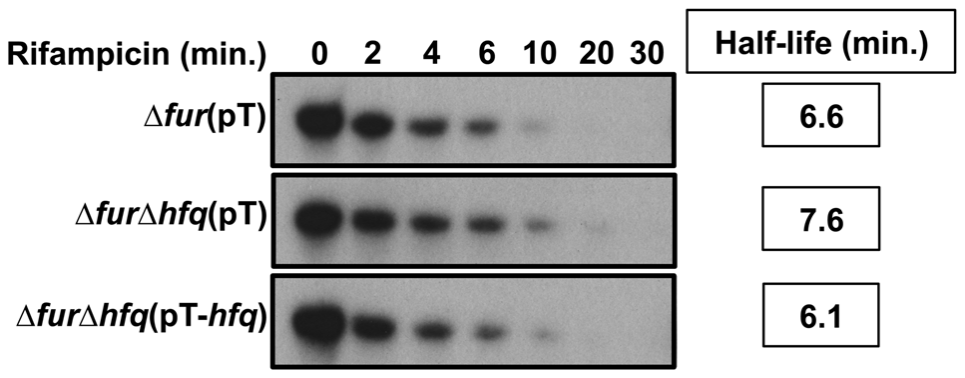
The half-life of HrrF is not dependent on Hfq. The 86-028NP*rpsL*Δ*fur*(pT), 86-028NP*rpsL*Δ*fur*Δ*hfq*(pT), and 86-028NP*rpsL*Δ*fur*Δ*hfq*(pT-*hfq*) strains were grown to mid-logarithmic phase in DIS at 37°C with shaking at 50rpm. Rifampicin was then added to a final concentration of 250 µM to inhibit transcription. At 0, 2, 4, 6, 10, 20, and 30 minutes after rifampicin addition total RNA was collected. RNA was probed via northern blot with an RNA probe specific for the 5′ end of HrrF. Blots were stripped and probed again with an RNA probe specific for 5S rRNA to serve as a loading control (data not shown). The calculated half-life of HrrF was approximately seven minutes in the 86-028NP*rpsLΔfur* strain. There was no significant difference in the calculated HrrF half-life in any of the strains tested. This indicated that the stability of *hrrF* was not dependent on the RNA chaperone Hfq.

To determine if Hfq has a role in the half-life of HrrF we grew 86-028NP*rpsL*Δ*fur*(pT), 86-028NP*rpsL*Δ*fur*Δ*hfq*(pT), and 86-028NP*rpL*Δ*fur*Δ*hfq*(pT-*hfq*) in DIS, followed by rifampicin addition to each culture to stop transcription. Culture aliquots were collected at specific intervals up to 30 minutes after rifampicin addition followed by RNA purification then by northern blot analysis using a probe specific for HrrF. HrrF transcript was quantified via densitometry to determine its half-life. The half-life of HrrF was approximately seven minutes in the 86-028NP*rpsLΔfur* strain. HrrF stability was not significantly altered in the absence of Hfq. Therefore, Hfq is not required for HrrF stability in NTHi strain 86-028NP*rpsL* (Fig. 5).

### HrrF target identification

RNA-Seq was performed using total RNA collected from strains 86-028NP*rpsL,* 86-028NP*rpsLΔfur and* 86-028NP*rpsLΔfurΔhrrF_L_* grown in DIS media. Comparison of the gene expression between the 86-028NP*rpsLΔfur* and 86-028NP*rpsLΔfurΔhrrF_L_* strains analyzed by RNA-Seq revealed that HrrF affects expression of six genes; *modA, modB, modC, asnA, asnC,* and *nrdB* (Table S4, GEO Series number GSE58891). The *modABC* locus, which encodes proteins involved in molybdate uptake, is downregulated by HrrF. In addition, HrrF positively affects gene expression of *asnA, asnC,* and *nrdB* encoding asparagine synthetase, the transcriptional regulator affecting *asnA* transcription, and the beta subunit of ribonucleotide-diphosphate reductase respectively (Table 1).

**Table 1:** HrrF targets identified via RNA-Seq.

			Fold Change		
NTHI no.	Gene	Function	Δ*furΔhrrF_L_*Δ*fur*	Δ*fur*86*rpsL*	Δ*furΔhrrF3'*Δ*fur*
NTHI2000	*modA*	molybdate-binding periplasmic protein	6.43	−14.08	−1.45
NTHI1999	*modB*	molybdate ABC transporter permease protein	3.14	−5.30	−1.06
NTHI1998	*modC*	molybdate transporter ATP-binding protein	2.48	−4.03	−1.00
NTHI0692	*asnA*	asparagine synthetase AsnA	−2.43	5.75	1.04
NTHI0691	*asnC*	DNA-binding transcriptional regulator AsnC	−2.30	3.64	1.21
NTHI1962	*nrdB*	ribonucleotide-diphosphate reductase subunit beta	−2.24	3.07	−1.16

### The ∼97bp HrrF sRNA is sufficient for activity

The HrrF_L_ transcript was observed at low levels when the *hrrF* region was maximally expressed (Fig. 3). Also, the 3′ extension of *hrrF_L_* is not present in most *Pasteurellaceae* strains suggesting that the ∼97bp *hrrF* is sufficient for regulating gene expression. We constructed a strain that lacked the *hrrF_L_* 3′ extended region, which deleted sequence between the first and second transcriptional terminators in the 86-*028NP hrrF* region. This strain was designated 86-028NP*rpsLΔfurΔhrrF3*′. The strain was grown in DIS and RNA-Seq analysis conducted as previously described. There was no difference in the expression of the six genes whose expression was affected by HrrF when the transcriptomes of 86-028NP*rpsLΔfur* (expressing the ∼260nt HrrF_L_) and 86-028NP*rpsLΔfurΔhrrF3*′ (expressing the ∼97nt HrrF) were compared (Table 1 column 6).

## Discussion

Understanding how bacterial pathogens respond to iron limitation has been extensively studied. Yet, for the *Pasteurellaceae* we know extremely little about how small regulatory RNAs are regulating gene expression and how these sRNAs affect gene expression in response to iron limitation. In this study we identified the single Fur-regulated sRNA in NTHi strain 86-028NP*rpsL.* Based on sequence similarity, this sRNA appears to be conserved among the *Pasteurellaceae.*

Identification of *hrrF* is the first report of an iron-responsive sRNA in any *Haemophilus* species. However there is a previous report of other iron-responsive sRNAs encoded by a *Pasteurellaceae* species. Work by Amarasinghe *et al.* reported the presence of four Fur-regulated sRNAs, JA01 through JA04, in *Aggregatibacter actinomycetemcomitans (Aa)* strain HK1651R [60]. BLAST analysis of these four sRNAs reveled that JA01 is only conserved among *Aa* strains. JA02 is present in both *Aa* and *P. multocida*. JA03 and JA04 are the most widely conserved of the four sRNAs and have orthologues across many *Pasteurellaceae* species. However, based on our RNA-Seq analyses, the JA03 and JA04 orthologous RNAs in strain 86-028NP*rpsL* are not expressed during mid-log growth and are not Fur-regulated. Furthermore, in strain 86-028NP*rpsL,* the JA03 and JA04 orthologues do not have predicted Fur boxes in close proximity. So while HrrF appears to be the most highly conserved Fur-regulated sRNA among the *Pasteurellaceae* it appears that some members may possess additional strategies to respond to low iron using sRNAs.

Northern blot analyses revealed the presence of two HrrF variants which are both Fur-regulated and iron-responsive. The variant with the greatest expression, *hrrF*, is ∼97nt in size, while, *hrrF_L_*, the ∼260nt variant is weakly expressed (Fig. 3). This observation is consistent with the presence of two putative Rho-independent terminators within the *hrrF* region. The first terminator begins 68bp downstream of the *hrrF* TSS. In the first terminator sequence, a U is present at nucleotide 89 resulting in a G:U wobble pair near the base of the stem. In the second terminator, a C is present at the corresponding position in the stem-loop resulting in a G:C base pair. The presence of U in the first terminator increases the ΔG free energy of the stem helix from -16.3 to -13.6 kcal/mol. This change in free energy may be enough to weaken the first stem-loop enough to allow occasional transcriptional read through and termination at the second stem-loop structure. As noted above, the 3′ extension *of hrrF_L_* is only present among a subset of *H. influenzae* strains and no other *Pasteurellaceae*. Under our experimental conditions, a mutant lacking sequences 3′ to the first terminator had a fully functional HrrF. It is unclear whether the large product, HrrF_L_, is advantageous to the bacterium under other conditions or simply not deleterious enough to be selected against and lost.

The importance of the 3′ region of *hrrF_L_* in 86-028NP is suggested by the corresponding region in HAE F3031. Strain F3031 contains an insertion of the *hafABCDE* (formerly *hif1*) locus between *hrrF* and *hpt.* This insertion is hypothesized to have arisen through a duplication event from elsewhere in the F3031 genome that was facilitated by the presence of intergenic dyad sequences thought to be important for the mobility of specific gene loci in *Haemophilus influenzae* species. [61], [62]. In F3031, the *hafABCDE* locus is inserted immediately upstream of the orthologous second terminator of *hrrF* in 86-028NP. Also, the sequence of the second Rho-independent terminator in 86-028NP is identical to the sequence of the first terminator in F3031. Therefore, the strongest terminator (as suggested by the predicted free energy of the stem-loops) appears first in strain F3031 suggesting that although F3031 contains the sequence of the extended *hrrF_L_* it may not be transcribed. It's possible that the 3′ extended sequence of the HrrF_L_ transcript present in 86-028NP is not functional and merely the result of genetic rearrangement which occurred in a common ancestor.

The alignments of HrrF among the *Pasteurellaceae* species (Fig. 2, Fig. S4) demonstrated that the majority of the represented species contained an *hrrF* sequence of ∼97nt in length. However, the more distantly related *Pasteurellaceae,* the non-*Haemophilus* species, contained a slightly longer *hrrF* sequence of about ∼126nt. The greatest region of sequence identity was within nucleotides 29-64 of HrrF (Fig. 2). The 100% sequence conservation across this region in multiple species suggests its importance in sRNA function.

Both an *in vitro* Fur pulldown and *in vivo* Fur box scrambling were employed to show that Fur binds to the *hrrF* promoter to regulate *hrrF* transcription. Individually scrambling the three Fur boxes upstream of *hrrF* demonstrated that Fur binds to and blocks transcription of *hrrF* at Fur box 1. Altering the sequences of Fur boxes 2 and 3 altered the transcription of *hrrF* preventing us from determining if Fur was able to bind in these regions.

The HrrF targets identified via RNA-Seq were *modA, modB, modC, asnA, asnC, and nrdB.* The *modABC* gene cluster is negatively regulated by HrrF, whereas *asnA, asnC, and nrdB* are positively affected by HrrF. For all HrrF targets identified, the extended 3′ portion present after the first terminator was dispensible for regulation (Table 1). A comparison of HrrF target regulation between strains 86-028NP*rpsL*Δ*furΔhrrF3′* and 86-028NP*rpsL*Δ*fur* (containing the full ∼260nt HrrF_L_) showed no difference in the regulation of HrrF indicating that the shorter ∼97nt HrrF transcript was capable of regulating target mRNAs as effectively as the full length ∼260nt HrrF_L_ transcript.

HrrF positively affected mRNA targets including *asnA, asnC,* and *nrdB.* It is unclear why a Fur-regulated sRNA like HrrF would upregulate *nrdB. nrdB* encodes the beta-subunit of the class Ia ribonucleotide-diphosphate reductase, an enzyme required for deoxyribonucleotide synthesis. This enzyme requires iron for normal function and therefore its upregulation during iron starvation is counterintuitive. *H. influenzae* encodes both the class Ia and class III ribonucleotide reductases expressed during aerobic or anaerobic conditions respectively [63]. Both of these enzymes require iron for function [63]. Absent from the 86-028NP genome is the class Ib ribonucleotide reductase which uses manganese as a cofactor and thus operates during conditions of iron starvation [38], [63], [64]. In the absence of a manganese-dependent ribonucleotide reductase, continued and elevated expression of *nrdB* may ensure that any available iron is bound by NrdB, the iron-containing portion of ribonucleotide reductase.

In addition to *nrdB,* HrrF also positively regulates *asnA* and *asnC. asnC* encodes a transcriptional regulator which activates *asnA* expression in *E. coli* [65]. *asnA* encodes asparagine synthetase important for the ammonia-dependent conversion of aspartate to asparagine [66]. Like *nrdB,* it is puzzling as to why asparagine synthesis would be upregulated during periods of low iron. Because HrrF affects *asnC* transcript levels, it is possible that positive regulation of *asnA* via HrrF is indirect and mediated through AsnC.

The *modABC* genes, encoding an ABC-transporter responsible for molybdate uptake, are negatively regulated by HrrF. (Fig. S5 contains the predicted binding interaction [67] between HrrF and *modB.*) In an effort to increase intracellular iron pools, numerous bacteria utilize an iron-responsive sRNA to downregulate non-essential iron-containing proteins [68]. This particular method of negative regulation by an iron-responsive sRNA was named iron sparing [14]. Although, HrrF is downregulating *modABC* expression, this ABC-transporter is not predicted to contain an iron cofactor. Therefore downregulation of *modABC* does not fit the iron-sparing model. However, molybdenum and iron metabolism are closely linked. Whenever molybdate is utilized as a protein cofactor it is always coordinated by a pterin structure named Moco (molybdate cofactor). The enzymes responsible for the first step in the biosynthesis of this pterin Moco contain iron-sulfur clusters [69]. Additionally, multiple enzymes that require a molybdenum cofactor have an iron component in the form of iron-sulfur clusters or heme [69]. Under conditions of iron limitation these iron-containing molybdoproteins would be non-functional. It is therefore tempting to speculate that downregulation of molybdate uptake during iron limitation is a mechanism to conserve energy. Import of molybdate during iron limitation may be an unproductive use of resources if the iron-containing molybdoproteins do not have the needed iron to function properly.

Lastly, we have shown that the RNA chaperone Hfq is not required for the steady-state stability or the half-life of HrrF. While uncommon, this finding is not unique. In *Yersinia pestis,* RyhB2 does not require Hfq for its stability [21]. Also in *N. meningitidis*, NrrF does not require Hfq for its stability [70] nor is Hfq required for the regulation of NrrF targets [70]. However, deletion of *hfq* in *N. meningitidis* caused an *in vitro* growth defect and disrupted the regulation of 45 genes including genes involved in metabolism and nutrient transport [70]. There is no clear link between requiring Hfq for sRNA stability and requiring Hfq for sRNA-mRNA interactions. However, we can draw parallels between the apparent lack for a role of Hfq in NrrF stability and gene regulation and the interactions of Hfq and HrrF in strain 86-028NP*rpsL.*

There is very limited study on the role of Hfq in NTHi strains. Hempel *et al.* showed that deleting Hfq in NTHi does not affect its ability to survive osmotic or detergent stresses [71]. Furthermore, an *in vitro* growth defect could only be observed when NTHi was grown in limited concentrations of human hemoglobin [71]. *hfq* mutant strains of NTHi exhibited no striking growth defects *in vitro*. However, in both a chinchilla model of OM or an infant rat model of bacteremia the *hfq* mutant strains were significantly impaired in persistence when in competition with the parent strains. [71].

Our own observations of the 86-028NP*rpsL*Δ*hfq* strain confirm the slight growth defect seen by others. We have not found any stress condition in which an *hfq* mutant strain survives more poorly than its parent strain. Even though there is still no clear role for Hfq in NTHi, Hempel *et al.* provide evidence suggesting that Hfq may be involved in nutrient acquisition. This is in agreement to what is known about the role Hfq has in gene regulation in *N. meningitidis.* Combined with our data on HrrF and from what is known for NrrF in *N. meningitidis* it is possible that in NTHi Hfq is not involved in HrrF-meditated gene regulation.

We have presented evidence of a newly identified Fur-regulated sRNA in NTHi. Furthermore, HrrF is conserved among the *Pasteurellaceae*. Although some *H. influenzae* strains contain an extended *hrrF* sequence, current evidence suggests that only the ∼97nt variant is biologically active. This work serves as the first step in identifying the HrrF regulon. Future work focused on a proteomics approach may expand the list of HrrF targets. Understanding how NTHi regulates its gene expression in response to iron limitation will provide clues as how to better combat this bacteria and prevent disease.

## Supporting Information

Figure S1 **Fold change in** ***hrrF*** **expression.** Strains were grown in DIS supplemented with 10 µg human hemoglobin/ml to mid-logarithmic phase. Total RNA was isolated from strains NTHi 86-028NP*rpsL* and NTHi 86-028NP*rpsLΔhrrF_L_.* Additionally total RNA was isolated from strain NTHi 86-028NP*rpsL* before and after chelation with 2,2′-bipyridine for 15min. Total RNA was then used in qRT-PCR with primers specific for *hrrF*. All threshold cycle (C_t_) values were normalized to the endogenous control *gyrA*. Relative quantitation was calculated from the median C_t_ value using ΔΔC_t_, and statistical significance was determined using the Student two-tailed t test. * indicates p-value < 0.05. ** indicates p-value <0.01.
(TIF)

Figure S2 ***hrrF*** **primer extension results. A.** Primer ES188 was labeled with a VIC fluorescent tag and used to generate cDNA from total RNA. The size of the resulting cDNA product was measured via capillary electrophoresis and determined to be 65nt in length. The top panel is the result from primer extension. The bottom panel is the result of a negative control in which no RNA was included in the reverse transcription reaction. The x-axis is cDNA length in nucleotides. The y-axis represents fluorescent intensity. **B.** Alignment of the fluorescent cDNA product shown in (A) with the results of a sequencing reaction of the *hrrF* promoter region using primer ES188. The x-axis is cDNA length in nucleotides. The y-axis represents fluorescent intensity.
(TIF)

Figure S3 **Layout of sequence similarities in the** ***pmbA-hpt*** **intergenic region.** A diagram of the sequence similarities and differences between the 12 representative *Pasteurellaceae* species
(TIF)

Figure S4 **Full alignment of the intergenic region between** ***pmbA*** **and** ***hpt*** **from selected** ***Pasteurellaceae.*** *hrrF* sequences from 12 *Pasteurellaceae* species were aligned using CLUSTALW. The ∼260nt *hrrF_L_* sequence occurs only in NTHi strains. In all strains queried there is a region in *hrrF* from position 29-64 that is 100% conserved suggesting a common function for this region. Nucleotides that match 86-028NP *hrrF* sequence are shaded black. The alignment is labeled with colored lines representing the following: purple = indicates the last 21nt and first 21nt of *pmbA* and *hpt* respectively, which flank the 5′ and 3′ end of *hrrF*; red =  *hrrF* sequence; blue = the 3′ extended region of *hrrF_L_* sequence; orange =  the first and last 21nt of the *hafABCDE* locus; green =  unique sequence 3′ of *hrrF.* The *hrrF* TSS is indicated with an asterisk. The *hrrF* Rho-independent terminators are marked with dotted lines. NTHi: nontypeable *Haemophilus influenzae,* HAE: *H. influenzae* biogroup *aegyptius*, *H.i.*: *Haemophilus influenzae, H.p.*: *Haemophilus parainfluenzae, A.a.* HK1651: *Aggregatibacter actinomycetemcomitans, A.a.* NJ8700: *Aggregatibacter aphrophilus, A.s.: Actinobacillus succinogenes, M.s.: Mannheimia succiniciproducens*
(TIF)

Figure S5 **Predicted binding interaction between HrrF and modB.** IntaRNA RNA interaction software was used to predicted the binding interaction of HrrF and its target modB. The seed region of HrrF is predicted to be between nucleotides 36 and 40 is indicated by a red line. HrrF is predicted to bind to the 3′ end of the *modB* coding region between nucleotides 549 and 553 with a net energy of -13.5 kcal/mol.
(TIF)

Table S1 **Bacterial strains and plasmids.**
(XLSX)

Table S2 **Primer sequences.**
(XLSX)

Table S3 **NTHi 86-028NP annotations with intergenic regions.**
(XLSX)

Table S4 **Gene expression analysis of 86-028NP** ***rpsL,*** **Δ** ***fur, ΔfurΔhrrF_L_,*** **and** ***ΔfurΔhrrF3′*** **strains.**
(XLSX)
