# Supplementary figures and images for: HrrF Is the Fur-Regulated Small RNA in Nontypeable Haemophilus influenzae
Source: PLoS One. 2014 Aug 26;9(8):e105644. doi: 10.1371/journal.pone.0105644 (PMC4144887; doi:10.1371/journal.pone.0105644)

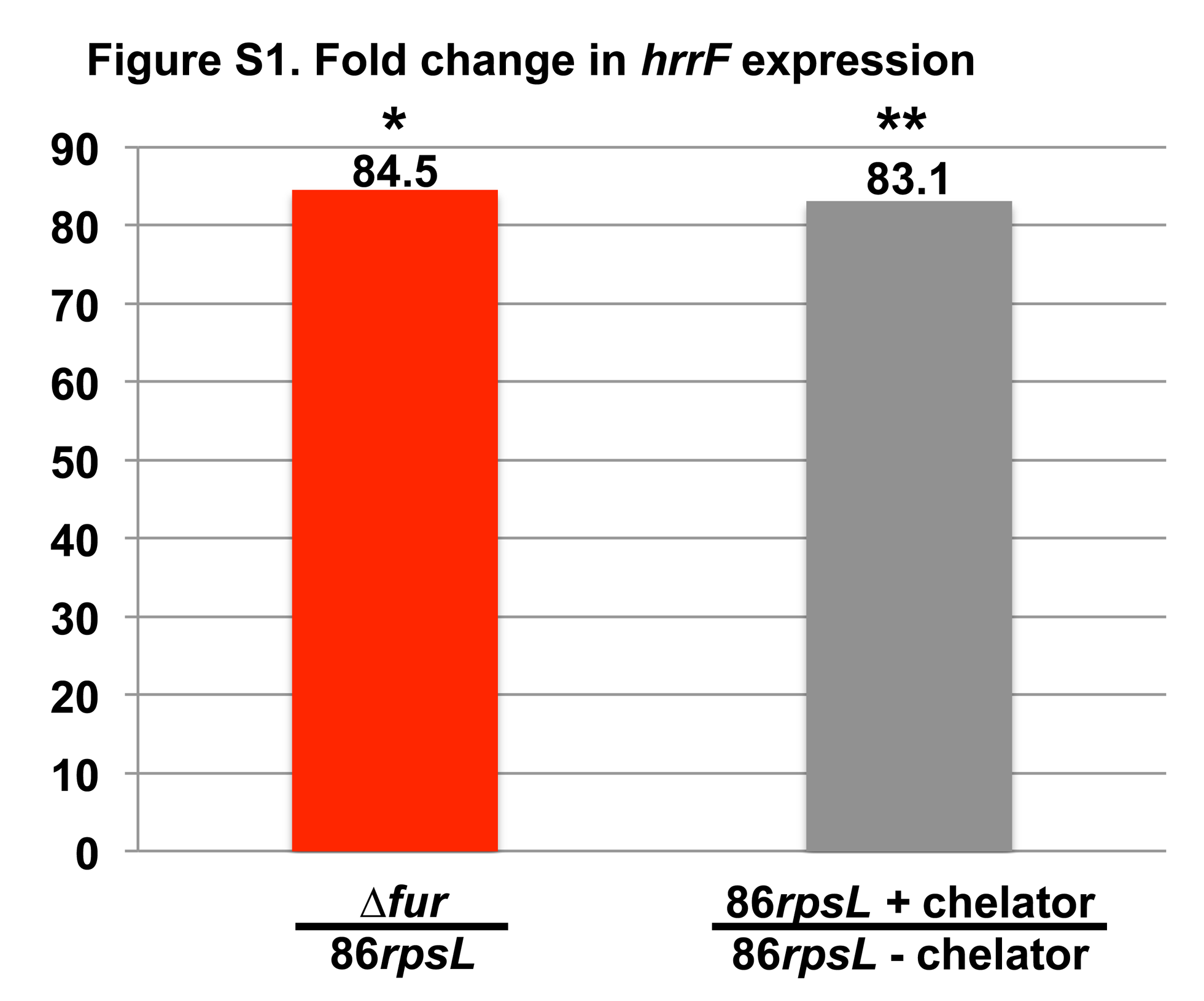

Supplement: Figure S1 — Fold change in hrrF expression. Strains were grown in DIS supplemented with 10 µg human hemoglobin/ml to mid-logarithmic phase. Total RNA was isolated from strains NTHi 86-028NPrpsL and NTHi 86-028NPrpsLΔhrrFL. Additionally total RNA was isolated from strain NTHi 86-028NPrpsL before and after chelation with 2,2′-bipyridine for 15min. Total RNA was then used in qRT-PCR with primers specific for hrrF. All threshold cycle (Ct) values were normalized to the endogenous control gyrA. Relative quantitation was calculated from the median Ct value using ΔΔCt, and statistical significance was determined using the Student two-tailed t test. * indicates p-value < 0.05. ** indicates p-value <0.01. (TIF) [file pone.0105644.s001.tif]

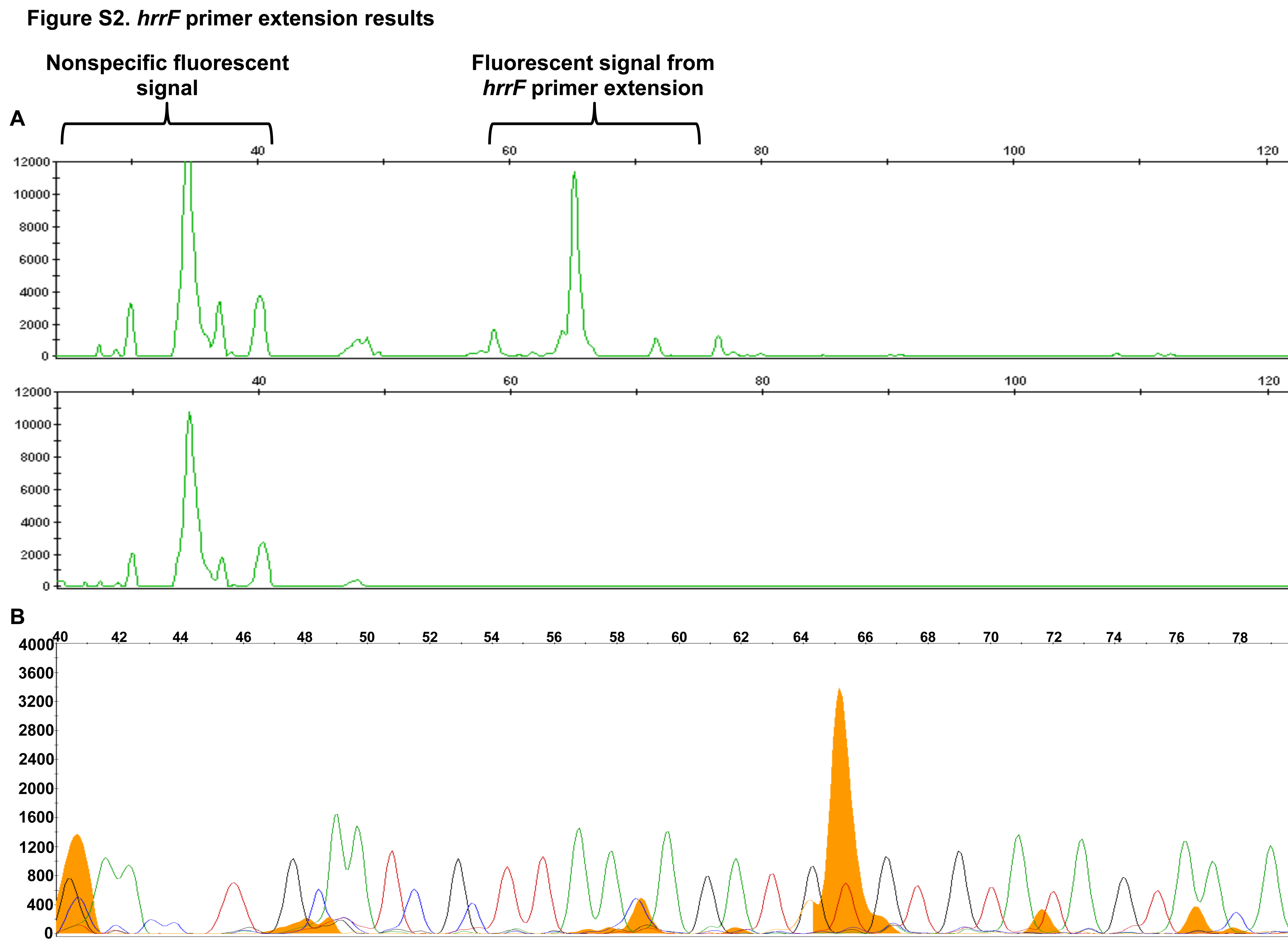

Supplement: Figure S2 — hrrF primer extension results. A. Primer ES188 was labeled with a VIC fluorescent tag and used to generate cDNA from total RNA. The size of the resulting cDNA product was measured via capillary electrophoresis and determined to be 65nt in length. The top panel is the result from primer extension. The bottom panel is the result of a negative control in which no RNA was included in the reverse transcription reaction. The x-axis is cDNA length in nucleotides. The y-axis represents fluorescent intensity. B. Alignment of the fluorescent cDNA product shown in (A) with the results of a sequencing reaction of the hrrF promoter region using primer ES188. The x-axis is cDNA length in nucleotides. The y-axis represents fluorescent intensity. (TIF) [file pone.0105644.s002.tif]

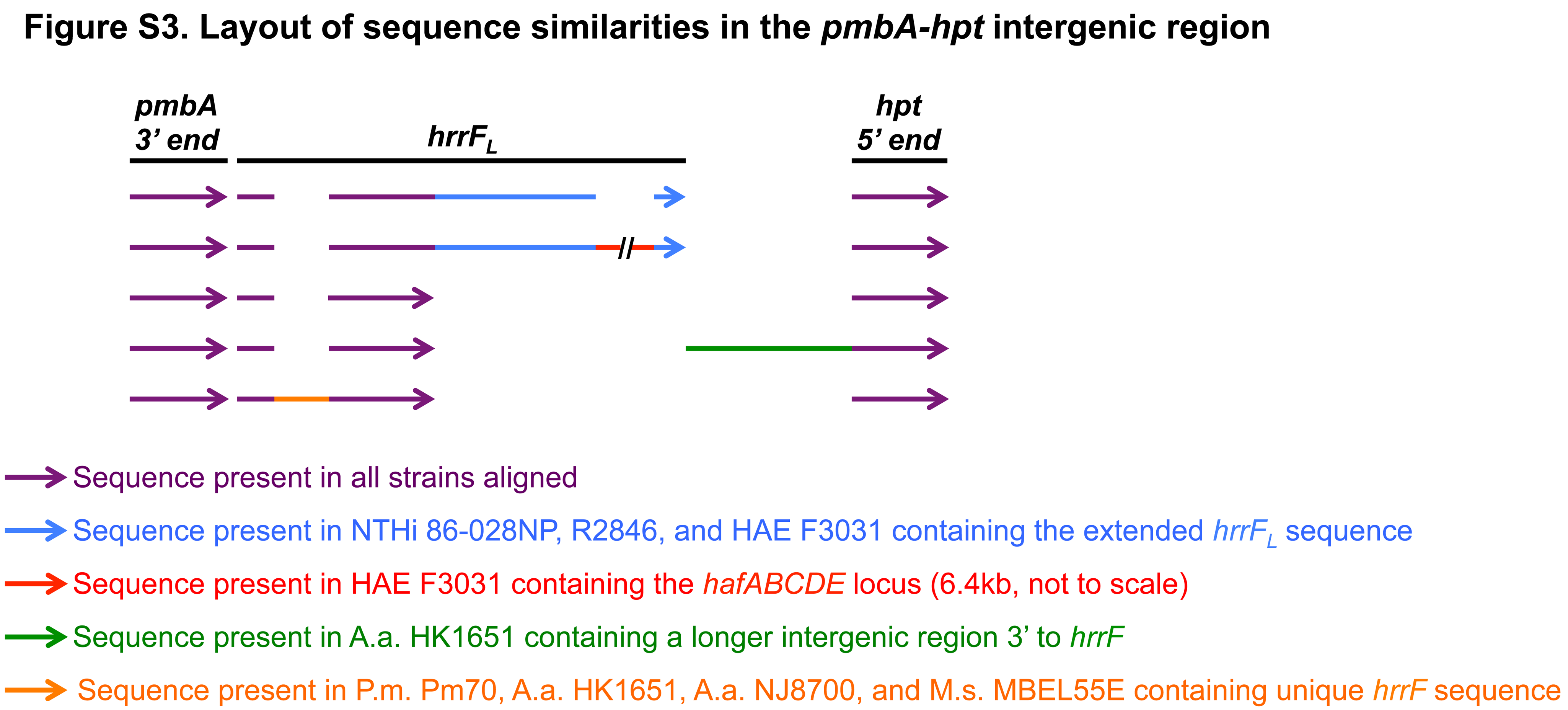

Supplement: Figure S3 — Layout of sequence similarities in the pmbA-hpt intergenic region. A diagram of the sequence similarities and differences between the 12 representative Pasteurellaceae species (TIF) [file pone.0105644.s003.tif]

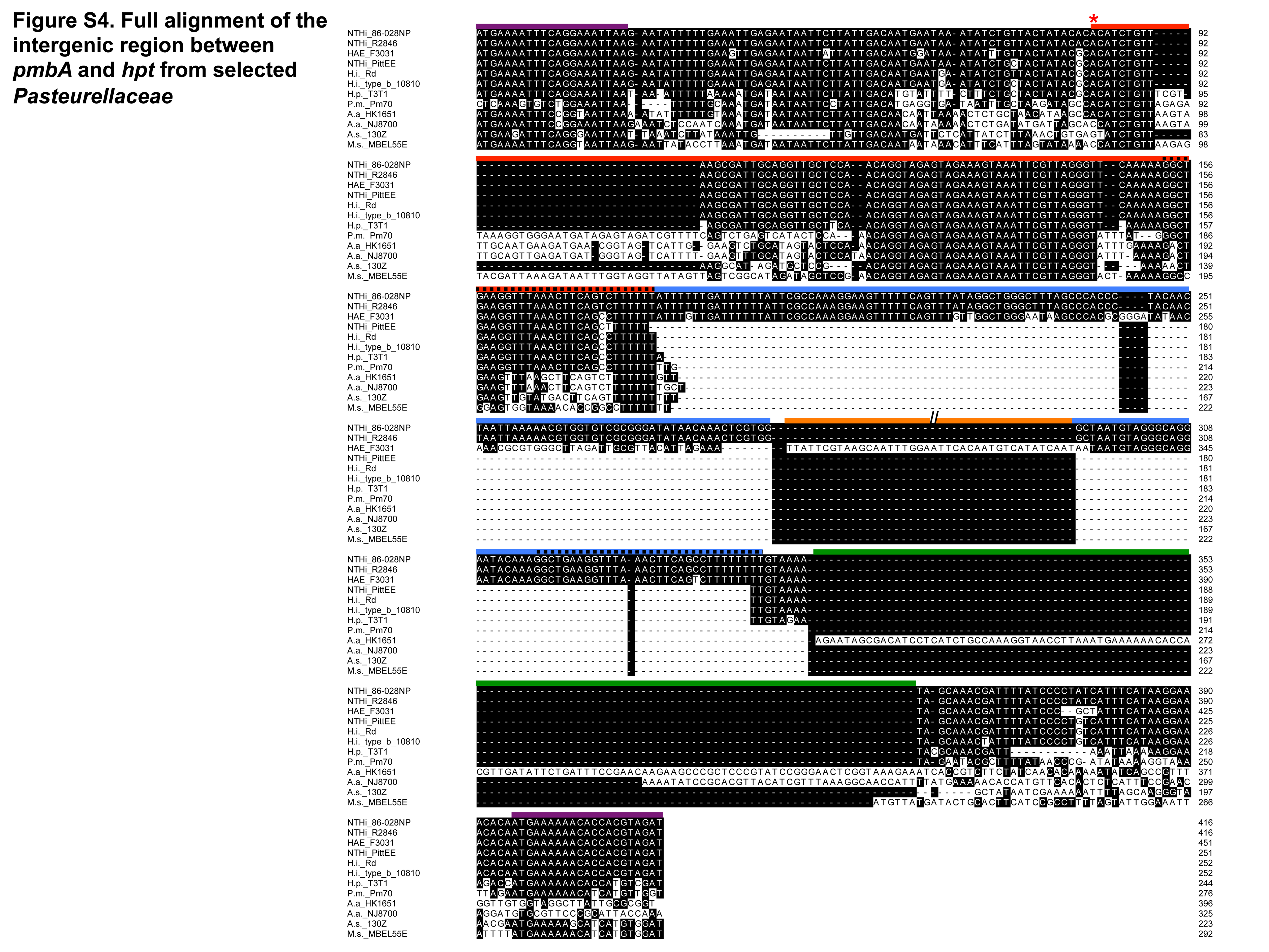

Supplement: Figure S4 — Full alignment of the intergenic region between pmbA and hpt from selected Pasteurellaceae. hrrF sequences from 12 Pasteurellaceae species were aligned using CLUSTALW. The ∼260nt hrrFL sequence occurs only in NTHi strains. In all strains queried there is a region in hrrF from position 29-64 that is 100% conserved suggesting a common function for this region. Nucleotides that match 86-028NP hrrF sequence are shaded black. The alignment is labeled with colored lines representing the following: purple = indicates the last 21nt and first 21nt of pmbA and hpt respectively, which flank the 5′ and 3′ end of hrrF; red = hrrF sequence; blue = the 3′ extended region of hrrFL sequence; orange = the first and last 21nt of the hafABCDE locus; green = unique sequence 3′ of hrrF. The hrrF TSS is indicated with an asterisk. The hrrF Rho-independent terminators are marked with dotted lines. NTHi: nontypeable Haemophilus influenzae, HAE: H. influenzae biogroup aegyptius, H.i.: Haemophilus influenzae, H.p.: Haemophilus parainfluenzae, A.a. HK1651: Aggregatibacter actinomycetemcomitans, A.a. NJ8700: Aggregatibacter aphrophilus, A.s.: Actinobacillus succinogenes, M.s.: Mannheimia succiniciproducens (TIF) [file pone.0105644.s004.tif]

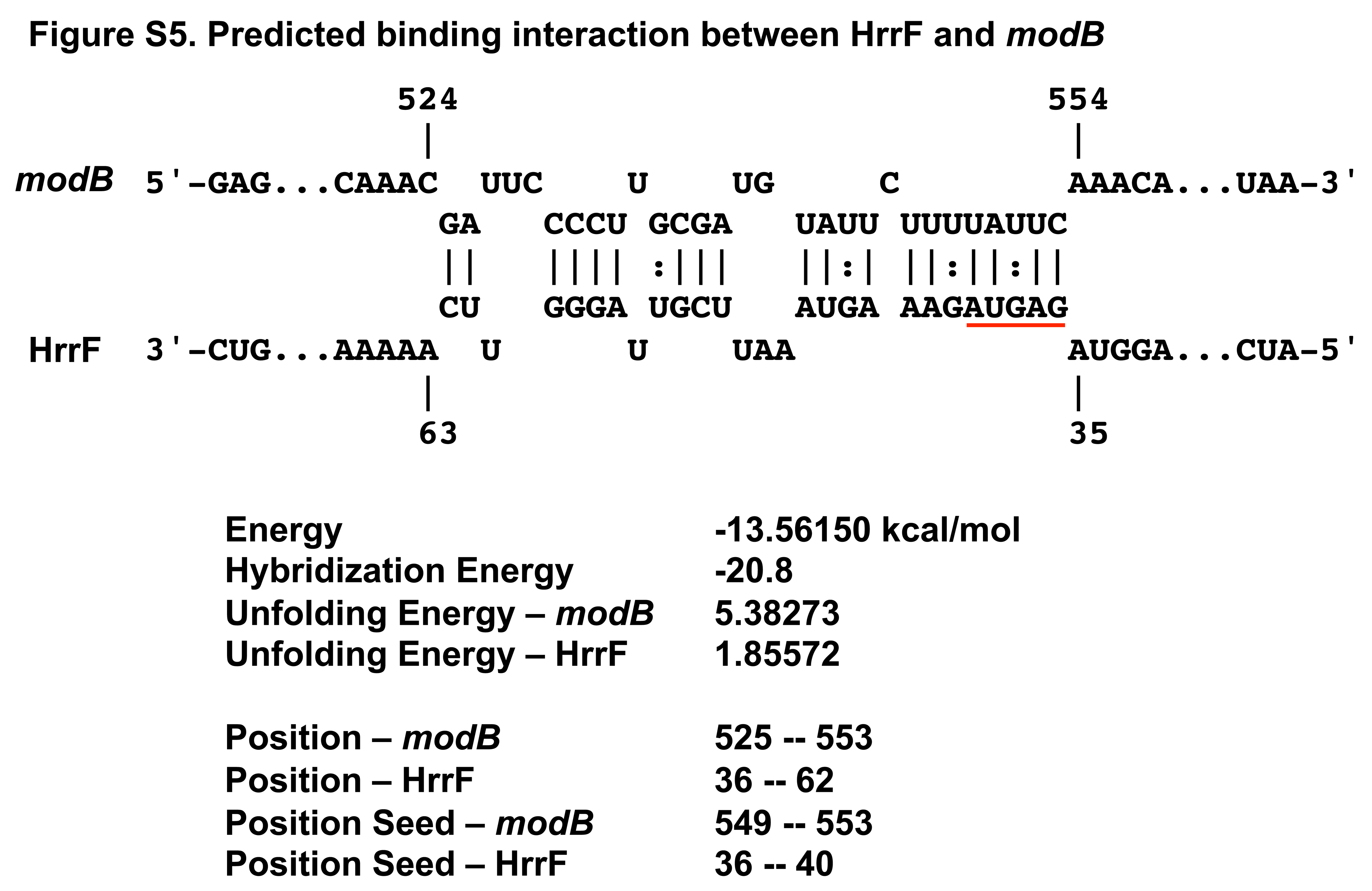

Supplement: Figure S5 — Predicted binding interaction between HrrF and modB. IntaRNA RNA interaction software was used to predicted the binding interaction of HrrF and its target modB. The seed region of HrrF is predicted to be between nucleotides 36 and 40 is indicated by a red line. HrrF is predicted to bind to the 3′ end of the modB coding region between nucleotides 549 and 553 with a net energy of -13.5 kcal/mol. (TIF) [file pone.0105644.s005.tif]
